# Crosstalk between the Rho and Rab family of small GTPases in neurodegenerative disorders

**DOI:** 10.3389/fncel.2023.1084769

**Published:** 2023-01-27

**Authors:** Shayan Nik Akhtar, Wyatt P. Bunner, Elizabeth Brennan, Qun Lu, Erzsebet M. Szatmari

**Affiliations:** ^1^The Harriet and John Wooten Laboratory for Alzheimer’s and Neurodegenerative Diseases Research, Brody School of Medicine, East Carolina University, Greenville, NC, United States; ^2^Laboratory of Neuroscience, Department of Physical Therapy, College of Allied Health Sciences, East Carolina University, Greenville, NC, United States

**Keywords:** Rho family, Rab family, small GTPases, neurodegeneration, intracellular trafficking

## Abstract

Neurodegeneration is associated with defects in cytoskeletal dynamics and dysfunctions of the vesicular trafficking and sorting systems. In the last few decades, studies have demonstrated that the key regulators of cytoskeletal dynamics are proteins from the Rho family GTPases, meanwhile, the central hub for vesicle sorting and transport between target membranes is the Rab family of GTPases. In this regard, the role of Rho and Rab GTPases in the induction and maintenance of distinct functional and morphological neuronal domains (such as dendrites and axons) has been extensively studied. Several members belonging to these two families of proteins have been associated with many neurodegenerative disorders ranging from dementia to motor neuron degeneration. In this analysis, we attempt to present a brief review of the potential crosstalk between the Rab and Rho family members in neurodegenerative pathologies such as Alzheimer’s disease (AD), Parkinson’s disease (PD), Huntington disease, and amyotrophic lateral sclerosis (ALS).

## Introduction

Despite their functional and morphological complexity, neurons share special requirements in intracellular trafficking between neuronal compartments involved in synaptic transmission, that include the soma, multiple dendrites, the axon, and the pre- and postsynaptic components (Hirokawa and Takemura, 2005; Villarroel-Campos et al., 2016). Induction and maintenance of neuronal asymmetry as well as the formation and conservation of neuronal networks require continuous membrane delivery, correct distribution of cell surface receptors, and constant remodeling of the actin and microtubule cytoskeletons (Cheng and Poo, 2012). Not surprisingly, dysfunctional membrane trafficking—mediated by Rab GTPases—and defective cytoskeletal dynamics—regulated by Rho GTPases—have been implicated in neurodegenerative disorders, including PD, AD, and ALS (Stankiewicz and Linseman, 2014; Kiral et al., 2018; Zhang et al., 2019, 2021; Oguchi et al., 2020; Homma et al., 2021).

As the largest branch of the Ras superfamily of small GTPases, different Rab proteins drive different sequential steps of the microtubule-based vesicular trafficking and sorting. As such, Rab GTPases are commonly used as vesicle and organelle markers within the endo- and exocytotic machinery (Kiral et al., 2018). Altered activity and up/downregulation of Rab proteins have been implicated in neurodegenerative disorders (Ginsberg et al., 2010, 2011; Yan et al., 2018). The roles of Rab GTPases in neurodegeneration can be either direct or indirect. For example, direct effects can be seen in neurodegeneration caused by mutations in rab genes and/or in the genes that encode Rab-associated proteins (Spinosa et al., 2008; Lesage et al., 2015), while alterations in Rab-dependent trafficking are associated with disease progression indirectly (Ginsberg et al., 2011; Lai et al., 2015).

Neurodegeneration also involves dysfunction of the actin and microtubule cytoskeletons that influence vesicular biogenesis and trafficking of vesicles and organelles between neuronal compartments (Mcmurray, 2000; Bernstein et al., 2011; Bamburg et al., 2021). Regarded as one of the central hubs for regulating cytoskeletal dynamics, Rho GTPases are critical mediators of cellular processes associated with neuronal polarity such as growth cone formation, dendritic spinogenesis, and axonal guidance (Li et al., 2002; Sin et al., 2002; Kalpachidou et al., 2019). Not only do proteins belonging to the Rab and Rho families of GTPases control trafficking between neuronal compartments, but there is also evidence for direct and indirect crosstalk between specific members of these families (Arrazola Sastre et al., 2020).

In the current review, we focus on several aspects of the potential crosstalk between members of small GTPases from Rab and Rho families in neurodegeneration. We first discuss the critical functions of Rho and Rab GTPases in neuronal structure and function in health and disease. Then we will examine the evidence supporting the crosstalk between Rho and Rab GTPases in selected disorders such as AD and PD. Finally, we will propose future research directions to address the physiological and clinical relevance of the Rho-Rab crosstalk in the context of neurodegeneration.

## Rho GTPases: structure and function in health and disease

Rho proteins belong to the Ras superfamily of small GTPases. This large superfamily has about 200 members divided into five families (Rho, Ras, Rab, Arf/Sar, and Ran), which are further divided into several subfamilies. In humans, the Rho family of GTPases contains 20 members and is divided into eight subfamilies (Rojas et al., 2012; Hodge and Ridley, 2016; Narumiya and Thumkeo, 2018). The most studied Rho proteins are RhoA, Rac1, and Cdc42, which are the focus of this review.

Rho signaling is known to regulate actin cytoskeleton dynamics associated with cellular polarity, motility, migration, and proliferation. They also have the ability to interact and respond to environmental stimuli (Heasman and Ridley, 2008; Hodge and Ridley, 2016; Song et al., 2019). Due to their ability to modulate the cytoskeleton, Rho GTPases are critical for cellular processes associated with neuronal morphology and physiology, including growth cone dynamics, axonogenesis, neuronal polarity, synaptic plasticity, and neuronal survival (Hall and Lalli, 2010; Stankiewicz and Linseman, 2014). Several studies have demonstrated the importance of Rho proteins in non-neuronal cell types in the brain, including their role in endothelial cell permeability and in astrocyte morphology (Wojciak-Stothard and Ridley, 2002; Zeug et al., 2018), further highlighting the importance of Rho GTPases in the nervous system.

**Figure 1:**
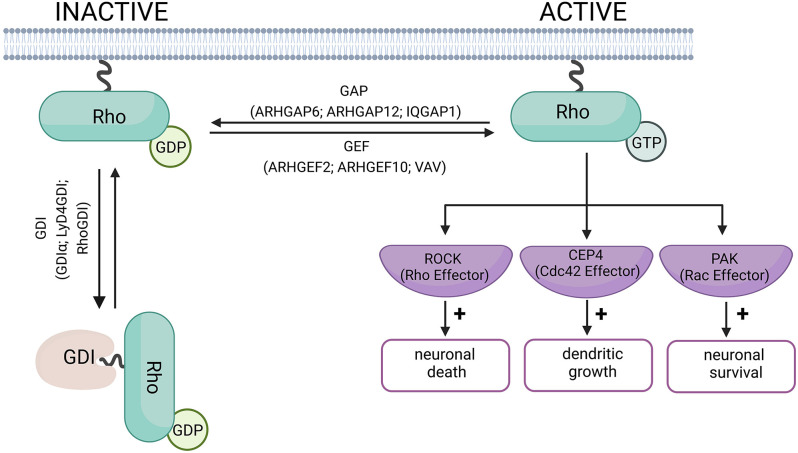
Schematic diagram of Rho-GTPase activation and inactivation. When Rho-GTPase is bound to GDP, it remains in its inactive form. Rho-GEF proteins mediate the switch from GDP to GTP, and activate the Rho-GTPase. Rho-GAP proteins hydrolyze the Rho-bound GTP to GDP, allowing the Rho-GTPase to return to its inactive form. The role of the GDI proteins is to prevent the exchange of GDP for GTP and keep the Rho-GTPase in its inactive form (Bos et al., 2007; Garcia-Mata et al., 2011). The examples of GEF and GAP proteins have been linked to neurodegenerative disorders and are discussed in this review. Examples of effector proteins and the cellular responses they mediate include Cdc42 effector protein-4, which promotes dendritic growth in neurons (Hou et al., 2022). Rho-induced ROCK activation can promote neuronal death, while Rac promotes neuronal survival (Stankiewicz and Linseman, 2014). Created with BioRender.com.

Rho GTPases are considered molecular switches due to their dual state of activation and inactivation (Figure 1). Briefly, in the inactive state, the GTPase is bound to Guanosine-5’-diphosphate (GDP) and in the active state, the GTPase is bound to Guanosine-5’-Triphosphate (GTP). The active and inactive forms of GTPase are controlled by three main types of proteins: Guanine nucleotide exchange factors (GEF, such as ARHGEF2, ARHGEF10, VAV), GTPase-activating proteins (GAP, such as ARHGAP6, ARHGAP12, IQGAP1), and Guanine nucleotide-dissociation inhibitors (GDI, such as GDIα, Ly/D4GDI, RhoGDI; Bos et al., 2007; Garcia-Mata et al., 2011). GEFs promote the dissociation of GDP and binding of GTP to GTPases (inactive to active Rho GTPase). GAPs, on the other hand, promote the enzymatic activity of the Rho GTPase resulting in the hydrolysis of GTP into GDP and inorganic phosphate (active to inactive Rho GTPase). The GDI does not allow the GDP to dissociate from the GTPase resulting in an inactive form of GTPase (Toma-Fukai and Shimizu, 2019).

### RhoA

#### Canonical function

RhoA is one of the members of the Ras Superfamily, which has been shown to modulate the actin cytoskeleton. RhoA through its downstream effectors such as RhoA-associated Kinase (ROCK) and Myosin Light Chin Kinase (MLCK) modulates actin stress fiber formation (Tapon and Hall, 1997).

#### Function in the nervous system

Although modulation of stress fibers is one of the most characteristic functions of RhoA GTPase, numerous studies highlight the role of RhoA in neurophysiological processes. For instance, in cerebellar granular neurons, RhoA was found to bidirectionally control axonal elongation either promoting it *via* the Rho-mDia pathway or repressing it *via* the Rho-ROCK pathway, depending on the concentration of stromal cell-derived factor-1 (SDF-1 Alpha) chemokine used for stimulation (Arrazola Sastre et al., 2020). Moreover, RhoA was shown to regulate the cellular and molecular processes underlying synaptic plasticity. Through its downstream effectors, ROCK or p160^ROCK^, RhoA promotes actin polymerization and long-term potentiation (LTP) in rat hippocampal neurons (Rex et al., 2009). Dominant negative form RhoA was found to promote dendritic arborization in Xenopus retinal ganglion cells (Newey et al., 2005). RhoA is also involved in neuronal survival, as shown by studies in which combined suppression of RhoA and Rac promoted neural stem/progenitor cell (NSPCs) survival in the injured spinal cord (Numano et al., 2009).

#### Involvement in neurodegeneration

RhoA signaling has been directly implicated in neurodegenerative diseases, including Alzheimer’s disease (AD), Picks disease (PiD), Parkinson’s disease (PD), and amyotrophic lateral sclerosis (ALS; Huesa et al., 2010; Aguilar et al., 2017; Schmidt et al., 2022). Dendritic spine loss, a hallmark of early neurodegeneration, is regulated by RhoA signaling. Treatment of cultured neurons with oligomeric beta amyloid (Aβo) activates the intracellular tyrosine kinase Pyk2 (PTK2B) which then phosphorylates and reduces the activity of the RhoGAP protein called GTPase regulator associated with focal adhesion kinase-1 (Graf1). Reduced RhoGAP activity results in increased RhoA activity which leads to dendritic spine loss (Lee et al., 2019).

In the human brain with AD or PiD, RhoA co-localizes with neurofibrillary tangles (NFTs), which are enriched with hyperphosphorylated Tau proteins (Huesa et al., 2010). Similarly, in the brain of Tg2576 AD model mice, RhoA level is reduced in the synaptic terminals, while dystrophic neurites show elevated RhoA expression (Huesa et al., 2010). Several studies evaluated neurodegeneration-associated changes in the level of RhoA effectors, such as ROCK1 and ROCK2. In human brains with mild cognitive impairment (MCI) and AD, ROCK1 levels are increased compared to non-AD brain matter (Henderson et al., 2016). This finding is further supported by studies using cellular models of AD: exposure of neurons to oligomeric Aβ42 increases ROCK1 and ROCK2 expression and induces phosphorylation of Lim Kinase 1 (LIMK) downstream of RhoA, while depletion of ROCK1 reduces toxic Aβ42 production (Henderson et al., 2016). In a study that looked at the effect of Aβ42 on BV2 microglial cells, Aβ42 treatment was shown to stimulate the production of reactive oxygen species (ROS), that was inhibited by blocking RhoA activity (Moon et al., 2013). ROCK involvement in PD progression was demonstrated by a study in which treatment with Fasudil, a small molecule ROCK inhibitor, promoted dopaminergic neuron survival and improved motor performance in a PD mouse model (Tonges et al., 2012). In an ALS mouse model, constitutive activation of Rho GTPase leads to the death of motor neurons *via* a mechanism that depends on Rho kinase or p160^ROCK^ activity (Stankiewicz et al., 2020).

### Rac1 GTPase

#### Canonical function

Activation of Rac1 is required for the formation of membrane ruffles and lamellipodia and involves the WAVE complex (Parri and Chiarugi, 2010). *Via* its downstream effectors such as PAK, p67phox, and MLK 2/3, Rac1 regulates the dynamics of actin cytoskeletal proteins, as well as transcription and nuclear signaling processes (Bosco et al., 2009).

#### Function in the nervous system

Rac1 protein belongs to the Rac GTPases subfamily which also includes Rac2, Rac3, and RhoG (Narumiya and Thumkeo, 2018). Rac1 is involved in establishing neuronal polarity and in neuronal survival. For example, the Rac/Cdc42 effector IQGAP3 is known to promote axonal outgrowth in hippocampal neurons (Wang et al., 2007). Rac1’s interaction with p21-activated kinase (PAK) promotes neuronal survival *via* inhibition of the Bcl-2-associated death promoter (Bad; Stankiewicz and Linseman, 2014). Relevant to ALS pathogenesis, NSC23766, a selective inhibitor of the Rac-specific GEFs Tiam1 and Trio, is sufficient to induce the death of embryonic stem cell (ESC)-derived motor neurons (Stankiewicz et al., 2020).

#### Involvement in neurodegeneration

In the human AD cortex, Rac1 expression decreases in parallel with increased Rac1 level in the plasma (Borin et al., 2018). Similarly, in the hippocampus of 3xTg-AD mice, Rac1 was reported to be active, but by 7 months of age, the total Rac1 level decreases (Borin et al., 2018). In mouse hippocampal neurons, pharmacological inhibition of Rac1 with NSC23766 reduced the expression of Amyloid Precursor Protein (APP) *via* decreasing the level of APP mRNA (Wang et al., 2009). Paradoxically, Manterola and colleagues demonstrated that in neuron-like cell lines (SN4741) and rodent primary neuron cultures, Rac1 activation by oligomeric Aβ42 involves Protein Kinase C (PKC), Phosphatidylinositol 3-Kinase (PI3K) and Phosphoinositol-Dependent Kinase (PDK1), causing loss of neurons. Pharmacological inhibition of PDK1 by OSU03012 resulted in Rac1 inactivation and subsequent inhibition of neuronal death (Manterola et al., 2013). Fibrillary Aβ was shown to interact with integrin receptors on microglial cells causing activation of a Rac1 GEF, Vav, which leads to enhanced Rac1 activity. Moreover, Vav^−/−^ microglial cells showed reduced production of ROS (reactive oxygen species) compared to Vav^+/+^ microglial cells (Wilkinson et al., 2006). Rac1 signaling is also involved in Tau pathology, which is another neuropathological hallmark of AD. In human neuroblastoma cells, Rac1 was found to indirectly increase Tau phosphorylation *via* translocation of SET, a protein phosphatase 2A (PP2A) inhibitor (Borin et al., 2018).

### Cdc42 GTPases

#### Canonical function

Cdc42 induces actin polymerization by interacting with the Wiskott–Aldrich syndrome protein (WASp) and neuronal-WASp (N-WASp). Cdc42 can also modulate the organization of microtubules by interacting with Cdc42-Interacting protein (CIP4). As a modulator of both actin and microtubule assembly, Cdc42 can induce both filopodia and membrane protrusions (Gerasimcik et al., 2015).

#### Function in the nervous system

There are three small GTPases belonging to the Cdc42 subfamily: Cdc42, RhoQ (TC10), and RhoJ (TCL; Narumiya and Thumkeo, 2018). In hippocampal neurons, Cdc42 was shown to promote actin polymerization *via* its effector N-WASP and the Arp2/3 complex. Through Cdc42, N-WASP induces filopodia and therefore modulates axon formation (Kitamura et al., 2003). By regulating cofilin, Cdc42 also plays an important role in establishing neuronal polarity. This is supported by findings that in Cdc42^−/−^ neurons, phosphorylation of cofilin increases, leading to abnormal axonal morphology (Garvalov et al., 2007; Hall and Lalli, 2010) Involvement of Cdc42 in synaptic plasticity, dendritic spine structural plasticity, and learning and memory was demonstrated by studies that show impaired synaptic plasticity and hippocampus-dependent memory in conditional Cdc42 KO mice (Kim et al., 2014).

#### Involvement in neurodegeneration

Several studies reported Cdc42 involvement in the progression of neurodegeneration. One study found that both AD and FTLD (frontotemporal lobar degeneration) are associated with elevated expression of Cdc42 in the frontal cortex, compared to age-matched controls (Saraceno et al., 2018). Several downstream target molecules have been proposed to mediate the Cdc42-dependent changes associated with neurodegeneration, including N-WASP. In cultured rat hippocampal neurons, Aβ exposure activated Cdc42 and Rac1, leading to increased F-actin formation (Mendoza-Naranjo et al., 2007).

As a functional regulator of Wnt signaling, one of the Cdc42 effectors is GSK3β (Etienne-Manneville and Hall, 2003), a kinase highly expressed in the brain with a critical role in neuropsychiatric and neurodegenerative disorders including AD (Llorens-Martin et al., 2014). *Via* phosphorylation of Tau, GSK3β contributes to NFT formation (Pelleieux et al., 2018). Interestingly, compounds identified from the Enamine’s screening collection can inhibit GSK3β-dependent Tau phosphorylation and reduce Tau aggregation in human neuroblastoma SH-SY5Y cells expressing Tau repeat domain (TauRD) with pro-aggregation mutation ΔK280 (Chiang et al., 2021). Based on these studies, it is postulated that pharmacological modulation of Cdc42 can be a potential avenue to better understand the role of Cdc42 signaling in neurodegeneration (Aguilar et al., 2017; Guiler et al., 2021).

## Rab GTPases: structure and function in health and disease

**Figure 2:**
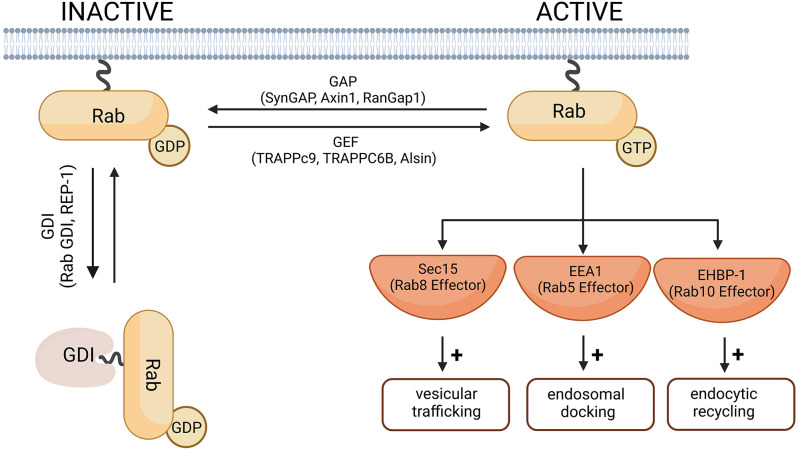
A schematic diagram of the Rab-GTPase activation and inactivation. In their inactive forms, Rab-GTPases are GDP-bound. The switch from GDP to GTP is mediated by Rab-GEF proteins and leads to Rab activation. Rab-GAP proteins hydrolyze the Rab-bound GTP to GDP and thus the Rab-GTPase returns to its inactive form. Cytosolic GDI proteins prevent the exchange of GDP for GTP and therefore keep the Rab protein in its inactive form. Representative Rab-GEF and Rab-GAP proteins linked to neurodegenerative disorders are given as examples and are further discussed in this review. Examples of effector proteins and the cellular responses they mediate include Sec 15 that mediates Rab8-dependent modulation of vesicular trafficking (Feng et al., 2012); EEA1, downstream of Rab5-dependent endosomal docking (Christoforidis et al., 1999), and EHBP-1 dowstream of Rab10-dependent endocytic recycling (Shi et al., 2010). Created with BioRender.com.

Human cells express more than 60 different Rab GTPases involved in the control of vesicle transport and formation (Stenmark and Olkkonen, 2001; Stenmark, 2009; Hutagalung and Novick, 2011). A universal function of all Rab GTPases is the control of membrane trafficking, Rab proteins being the key regulators of intracellular transport (Guadagno and Progida, 2019). Like other small GTPases, Rab proteins cycle between a GTP-bound active and GDP-bound inactive form (Figure 2). The switch between active and inactive forms is tightly regulated *via* effector proteins, which can cause a conformational change in Rab GTPases by interacting with the switch I and II regions (Stenmark, 2009). The activity of the GTPases is regulated primarily by GAPs (such as SynGAP1, Axin1, RanGap1), which converts the GTPase to its inactive form by catalyzing the hydrolysis of GTP to GDP. Rab proteins in their inactive form are stabilized by GDIs (such as Rab GDI, REP-1), which bind to inactive Rab and prevents its reactivation by GEFs (such as TRAPPc9, TRAPPC6B, Alsin). GEFs catalyze the activation of Rab GTPases by promoting GDP to GTP exchange (Stenmark and Olkkonen, 2001). GEFs and GAPs play an important role in the temporal control of Rab GTPases and in regulating how long they remain active (Wandinger-Ness and Zerial, 2014). Rab-GAPs can enhance the intrinsic activity of GTPases allowing for rapid hydrolysis of their GTP to GDP. For example, Rab5, when active, simultaneously recruits TBC-4, a Rab10 GAP, to sharply reduce Rab10 activity (Sasidharan et al., 2012). Conversely, DENND4 as a GEF for Rab10, converts Rab10 from its GDP-bound to GTP-bound form enabling it to participate in cellular functions such as the translocation of GLUT4 (Sano et al., 2011).

An individual Rab protein can have multiple functions through interactions with different effectors, such as motor proteins, coat proteins, tethering complexes, and Snares (Hutagalung and Novick, 2011). Downstream effectors direct Rab signaling towards different stages of membrane transport including vesicle budding, tethering, and mobility (Hutagalung and Novick, 2011). Coat proteins, such as COPI and COPII facilitate the formation of cytosolic coat complexes around the cargo (Bonifacino and Glick, 2004). Rab GTPases also contribute to ensuring the correct coat recruitment to the intracellular membrane. One example of Rab GTPases contributing to vesicle budding can be seen in Rab9 signaling. This Rab enables the recycling of mannose-6-phospate receptors (M6PRs) from the late endosomes to the trans-Golgi network. The tail of M6PRs is recognized by TIP47, a sorting adaptor and an effector of Rab9. The interaction between Rab9 and M6PRs recruits TIP47 to late endosome membranes and increases the affinity of TIP47 for its cargo, thus facilitating M6PR sorting into late endosomal recycling buds (Carroll et al., 2001).

Particularly in neurons, this diverse set of interactions is critical for compartmentalized protein trafficking that is crucial for the induction and maintenance of neuronal polarization and synaptic structure and function. Below, we provide a brief overview of the Rab proteins that have been associated with neurodegenerative disorders.

### Rab1 GTPases

#### Canonical function

Although the main function of Rab1 is to regulate the ER-to-Golgi transport, it is also implicated in signaling pathways associated with other cellular processes such as nutrient signaling, integrin-dependent cell migration, and autophagy. Rab1 signaling is unique among small GTPases, as they do not activate their effectors; instead, they regulate their targeting and formation of active signaling complexes on their respective membranes (Yang et al., 2016).

#### Function in the nervous system

Rab1 is involved in neuronal development, particularly in the formation of neurites (Villarroel-Campos et al., 2014) Rab1 tubules are able to move bidirectionally between the central body of the neuron and neurites that connect to the central Golgi and ER exit sites (Saraste, 2016).

#### Involvement in neurodegeneration

Rab1 dysfunction has been implicated in the pathogenesis of PD. In human neuroblastoma cells, α-synuclein overexpression disrupts Rab1A activity resulting in a hindered ability of autophagosome synthesis. Moreover, in a rodent model of PD, increasing Rab1 expression reduces α-synuclein-mediated Golgi fragmentation, prevents the death of dopaminergic neurons, and ameliorates PD-like motor deficits (Winslow et al., 2010).

### Rab3 GTPases

#### Canonical function

Members of the Rab3 subfamily (Rab3A, Rab3B, Rab3C, and Rab3D) are primarily restricted to neurons, but the A, B, and C isoforms can be found in endocrine tissue as well. The D isoform is expressed in adipose tissue, exocrine glands, and the endocrine pituitary. The GDP/GTP exchange cycle of Rab3A is required for the Ca^2+^-regulated exocytosis to occur (Schluter et al., 2004).

#### Function in the nervous system

Rab3 GTPases are predominantly localized to the presynaptic active zone where they regulate the priming and docking of synaptic vesicles (Vieira, 2018). Interestingly, deleting one or two of these genes in mice had no phenotype; however, triple or quadruple knockouts resulted in lethality if Rab3A was part of the deletion (Schluter et al., 2006).

#### Involvement in neurodegeneration

Several findings directly implicate Rab3 in synaptic dysfunction associated with Parkinson’s disease (Chen et al., 2013). Similar to Rab1, Rab3 overexpression partially rescues the α-synuclein-induced dysfunction of dopaminergic neurons (Gitler et al., 2008). In another study, Rab3 was shown to directly interact with α-synuclein. It was reported that Rab3A recycling machinery regulates the membrane binding of α-synuclein (Chen et al., 2013).

### Rab4 GTPases

#### Canonical function

Rab4 is involved in cell secretion and metabolism *via* regulating the vesicular trafficking between the recycling and degradation pathways (Conti et al., 2009). Transfer of the vesicular cargo from early endosomes to the recycling endosomes requires Rab4 recruitment to the intermediate sorting endosomes (Grant and Donaldson, 2009).

#### Function in the nervous system

In neurons, Rab4 is involved in axonal outgrowth *via* endosome recycling. In a study using Xenopus neurons, Rab4 downregulation resulted in axonal elongation (Falk et al., 2014).

#### Involvement in neurodegeneration

Expression levels of Rab4 were found to be increased in human brains with MCI as well as in late onset AD brain matters (Cataldo et al., 2000; Ginsberg et al., 2011). These findings are supported by studies using cellular and murine models of AD. For example, the level of Rab4 significantly increases in neurons that carry AD-relevant mutations in the *PSEN1* gene and in the brain of 3xTg-AD model mice as the disease progresses (Soejima et al., 2013). CD2AP, a scaffolding protein for the actin cytoskeleton and a Rab4a effector, is another potential link between Rab4a and AD (Monzo et al., 2005). Loss of function mutation of CD2AP leads to enhanced Aβ production (Guimas Almeida et al., 2018; Van Acker et al., 2019) and increased Tau-induced neurotoxicity (Shulman et al., 2014).

### Rab5 GTPases

#### Canonical function

Rab5 GTPase plays a critical role in clathrin-mediated endocytosis and endosomal maturation (Semerdjieva et al., 2008). Rab5 also helps mediate the kinetics of nuclear membrane disassembly and plays an important role in the morphology of the peripheral ER (Audhya et al., 2007).

#### Function in the nervous system

In the *Drosophila* neuromuscular junction, Rab5 promotes synaptic endosomal integrity (Wucherpfennig et al., 2003). In mice, Rab-5-dependent endosomal sorting regulates the size uniformity of synaptic vesicles (Shimizu et al., 2003).

#### Involvement in neurodegeneration

Rab5 has been implicated in neurodegeneration, particularly in AD and HD. In the human brain with late onset AD, the size of Rab5 positive early endosomes is significantly larger than in the healthy non-AD brain (Cataldo et al., 2000). In murine models of AD and in the brain of mice overexpressing Rab5, it has been shown that overactivation of Rab5 is sufficient to drive AD-like neurodegeneration and cognitive decline (Pensalfini et al., 2020). Mechanistically, Rab5 involvement in AD might be due to its association with early endosomes (EE) that are upregulated and enlarged in the aging neurons, partly due to increased APP endocytosis (Burrinha et al., 2021). Consequently, Rab5 upregulation in mature neurons significantly increases intracellular Aβ42 production and induces synaptic dysfunction (Burrinha and Guimas Almeida, 2022). Studies using cellular models of HD found that Rab5 interacts with the Huntingtin-HAP40 complex that regulates the activity of early endosomes (Pal et al., 2006; Ravikumar et al., 2008). Disruption of the Rab5-Huntingtin-HAP40 complex leads to a reduction in endosomal motility (Pal et al., 2006).

### Rab6 GTPases

#### Canonical function

Rab6 plays a role in the control of retrograde vesicle transport from the Golgi apparatus toward the endoplasmic reticulum (ER; Opdam et al., 2000). Rab6 is also critical for basement membrane formation (Homma et al., 2019).

#### Function in the nervous system

Rab6 is highly expressed in the brain where it has predominantly pre-synaptic distribution and plays a role in the control of retrograde vesicle transport from the Golgi apparatus toward the ER (Opdam et al., 2000). Studies in *Drosophila* demonstrated that Rab6 plays a role in synaptic specificity *via* activation of Ric1 homologue-Rab6-N-Cadherin pathway (Tong et al., 2011). In a more recent study, Rab6 was identified in cargo delivery to synapses, a function that requires ELKS1 (Nyitrai et al., 2020).

#### Involvement in neurodegeneration

Rab6 might be involved in neurodegenerative disorders, particularly AD (Elfrink et al., 2012). Elevated Rab6 expression has been found in the AD brain, as well as in cells carrying *PSEN1* gene mutations (Scheper et al., 2004, 2007). Early studies of the role of Rab6 in AD pathogenesis suggest that Rab6 can influence whether APP is sorted into the α-secretase pathway or the amyloidogenic β-secretase pathway. In HEK 293 cells co-expressing a defective GTP-binding Rab6 mutant and APP, secretion of the soluble APP alpha increased, whereas production of the peptides derived from the amyloidogenic processing pathway was either slightly inhibited or unaffected (Mcconlogue et al., 1996).

### Rab8 GTPases

#### Canonical function

Members of the Rab8 subfamily regulate cell shape and protrusion formation (Hattula et al., 2006; Peranen, 2011). Activation of Rab8 positively regulates the formation of lamellipodia and filopodia, whereas its inhibition negatively impacts these structures (Peranen et al., 1996).

#### Function in the nervous system

Rab8 proteins are involved in the transport of GluA1-AMPA receptors from the ER-Golgi network to the postsynaptic membrane (Gerges et al., 2005; Hausser and Schlett, 2019). Inhibition of neuronal Rab8 compromises membrane transport, resulting in impaired neurite outgrowth. Mechanistically, Rabin8, a shared GEF between RAB8, RAB10, and RAB11 is central to control of neurite outgrowth (Homma and Fukuda, 2016).

#### Involvement in neurodegeneration

Rab8 involvement in AD neurodegeneration is supported by a study showing a significant reduction in PSEN1 mutated cells compared to cells that expressed normal PSEN1 (Kametani et al., 2004). Rab8 has also been implicated in PD. In a cellular PD model, gain-of-function mutations in LRRK2 caused lysosomal sequestration of endogenous Rab8a, which was reversed by pharmacological inhibition of LRRK2 (Mamais et al., 2021).

Rab8 also has HD implications. In a *Drosophila* model of the disease, increased Rab8 level provided neuroprotection against mutant Huntingtin (HTT) *via* reducing the accumulation of downstream soluble toxic species (Delfino et al., 2020). Another member of the Rab8 GTPase subfamily, Rab10, has been strongly implicated in AD pathogenesis (Yan et al., 2018; Tavana et al., 2018). In cellular models of AD, Rab10 silencing was shown to significantly decrease the production of toxic Aβ42 (Ridge et al., 2017). Genetic studies of elderly individuals at high-risk for AD (*APOE ε4* carriers) who escaped the disease, identified a rare Rab10 variant as a potential provider of molecular resilience against AD (Ridge et al., 2017).

### Rab11 GTPases

#### Canonical function

Proteins belonging to the Rab11 subfamily regulate the trafficking of the recycling endosomes and early endosomes to the trans-Golgi network (Wilcke et al., 2000).

#### Function in the nervous system

Rab11 plays a major role in dendrite outgrowth and is also involved in receptor trafficking and recycling (Dong et al., 2010). It is critical for the activity-dependent delivery of GluA-containing AMPA receptors to the synapses (Park et al., 2004).

#### Involvement in neurodegeneration

Several studies implicate Rab11 in neurodegeneration (Bhuin and Roy, 2015). Rab11 was shown to be neuroprotective in a *Drosophila* model of PD by restoring the size of synaptic vesicles and rescuing α-synuclein-induced locomotor impairment (Breda et al., 2015) as well as by protecting against mitochondrial dysfunction induced by *parkin* mutations (Rai and Roy, 2022). In a *Drosophila* model of HD, overexpression of Rab11 rescued synaptic dysfunction and behavioral deficits (Steinert et al., 2012). Similarly, in mouse models of HD, elevating Rab11 activity was sufficient to reduce oxidative stress and neuronal loss (Li et al., 2009, 2010).

RNAi screening of all human Rab proteins that regulate membrane trafficking using cells with robust Aβ and sAPPβ production (primary neuron cultures derived from AD model mice and cell lines with stable expression of APP with Swedish mutation) identified Rab11 as a key regulator of Aβ production. The molecular mechanism involves Rab11-mediated recycling of BACE-1 to EE, followed by enhanced Aβ production (Udayar et al., 2013).

## Interactions of Rho and Rab proteins in modulation of neuronal structure and function

### Interaction between Rab and Cdc42 GTPases

Cytoskeletal rearrangement and membrane trafficking play an important role in neuronal migration and establishment of dendritic and axonal morphology (Kawauchi et al., 2010; Kawauchi, 2011; Villarroel-Campos et al., 2016). Cdc42 and Rab GTPases are known to regulate the remodeling of the cytoskeleton and to control membrane trafficking, respectively. Moreover, Cdc42 assists in neuronal migration and neuronal progenitor proliferation (Kawauchi, 2011). *Via* its interaction with Lis1 and IQGAP1, Cdc42 can potentially enhance neuronal migration and promote axon specification by allowing the activation of the PAR polarity complex consisting of atypical protein kinase C (aPKC), Par3, and Par scaffolding protein (Schwamborn and Puschel, 2004; Kholmanskikh et al., 2006; Lee et al., 2013).

Among Rab proteins, Rab5 was reported to assist in neuronal migration. As such, Rab5 deletion blocks neuronal migration in the mouse cortex (Kawauchi et al., 2010; Kawauchi, 2011). Similarly, suppression of Rab11 also resulted in delayed neuronal migration (Kawauchi, 2011). Both exocytic and endocytic pathways are critical for neuron differentiation, elongation of neuronal processes, and their migration (Kawauchi, 2011; Urrutia et al., 2021). Hence Cdc42 and Rab GTPases might crosstalk in modulating neuronal morphology, migration, and polarity (Kawauchi, 2011; Villarroel-Campos et al., 2016; Urrutia et al., 2021). Cdc42 induces filopodia formation through its effector protein Wiskott-Aldrich syndrome protein (WASP). The homolog of WASP in *Caenorhabditis elegans* (*C. elegans*), ***wsp-1***, was shown to play an important role in synaptic transmission at the neuromuscular junction (Zhang and Kubiseski, 2010). Mutations in the wsp1 gene lead to increased sensitivity to the neuromuscular acetylcholinesterase inhibitor aldicarb (Zhang and Kubiseski, 2010). This sensitivity was reduced by ***WSP-1*** rescue. Moreover, the reduction in sensitivity was due to WSP-1 interaction with the WSP-1 Cdc42/Rac interacting domain (CRIB). The WSP-1 protein was found to be co-localized with Rab proteins in the presynaptic terminal (Zhang and Kubiseski, 2010). The close localization between WSP-1 (a Cdc42 effector protein) and Rab-GTPases suggest that there could be a possible interaction between Cdc42 and Rab-GTPases in the modulation of synaptic transmission.

**Figure 3:**
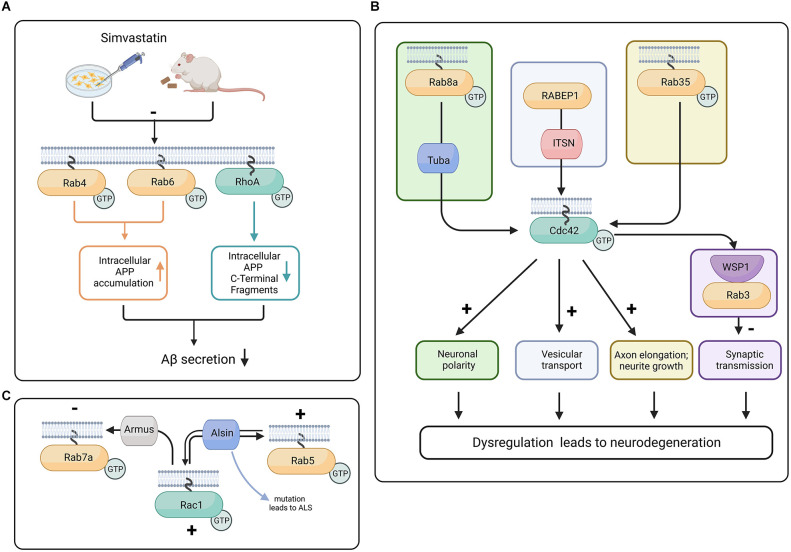
Proposed crosstalk between Rab and Rho GTPases in progression of neurodegeneration. **(A)** In mouse and N2a cells, the treatment with Simvastatin reduces isoprenylation of RhoA, Rab4, and Rab6. The inhibition of isoprenylation of Rho and Rab proteins can *potentially* reduce Aβ secretion and therefore have neuroprotective effects (Ostrowski et al., 2007, 2016). **(B)** Tuba activates Cdc42 downstream of Rab8a, promoting neuronal polarity (Urrutia et al., 2021), whereas Rab35 activity increases Cdc42 activity and promotes neurite growth (Chevallier et al., 2009). In *C. elegans*, the Cdc42 effector protein WSP-1 is colocalized with Rab3 at the synaptic terminal and is responsible for stabilizing the actin cytoskeleton and restraining the abnormal release of vesicles (Zhang and Kubiseski, 2010). Intersectin (ITSN) interacts with the effector protein of Rab GTPase called RABEP1 and has a GEF domain for Cdc42, *potentially* facilitating vesicular transport. Because of its GEF and scaffolding ability, ITSN is a potential link between Rab and Rho GTPases (Herrero-Garcia and O’Bryan, 2017). Dysregulation of neuronal polarity, deficient synaptic transmission, and defects in vesicular transport are major contributors to neurodegeneration (Dubey et al., 2015; Subramanian et al., 2020; Blackstone et al., 2021). **(C)** Rac1 influences Rab7a function through Armus, a Rab7a GAP (Margiotta and Bucci, 2019). Armus recruits and inactivates Rab7a. Alsin has the capability to act as GEF for both Rab5 and Rac1 (Topp et al., 2004; Chandran et al., 2007). Created with BioRender.com.

Additionally, in a study using mouse hippocampal neurons and N1E-115 neuroblastoma cell line, it was demonstrated that Tuba, a Cdc42 GEF, was required for neuronal polarization, as shRNA knockdown of Tuba increased the number of unpolarized neurons as well as decreased axonal length (Urrutia et al., 2021). Rab8a activity results in an increased density of Tuba proteins at the distal end of the axon. In the neuroblastoma cell line, it was demonstrated that Rab8a activity promotes Cdc42 activation through Tuba. Tuba and Rab8a also regulate the morphology of migrating neurons (Urrutia et al., 2021; Figure 3B). Aside from Rab8a, Rab35 has also been demonstrated to modulate Cdc42 activity in neurons, as Rab35-induced neurite growth dependent on Cdc42 and Rac1. The constitutively active form of Rab35 enhanced Cdc42 activity in BHK fibroblast cells, and a dominant negative form of Rab35 reduced the activity of Cdc42 (Villarroel-Campos et al., 2016).

Moreover, the cholesterol biosynthesis pathway has intermediate isoprenoid compounds such as farnesyl pyrophosphate (FPP) and geranylgeranyl pyrophosphate (GGPP). FPP (a 15 carbon chain) and GGPP (a 20 carbon chain) are covalently bound to the C-termini of Rho and Rab family proteins (known as protein isoprenylation), assisting these proteins during their relocation to the plasma membrane. Levels of FPP and GGPP have been reported to increase in the aging rodent brain as well as in the human AD brain (Hottman and Li, 2014). In studies using N2a cells, Simvastatin was shown to inhibit isoprenylation of Cdc42, RhoA, and Rab (Ostrowski et al., 2016; Figure 3A).

Another potential protein linking the modulation of cytoskeletal and vesicular systems is Intersectin (ITSN), a multidomain protein with scaffolding and GTPase GEF domains. ITSN has the ability to modulate Cdc42, Rac, and Ras GTPases. In the case of Ras, when it loses its GDP and before accepting GTP, it is in an in-between state referred to as “nucleotide free ras” (nfRas). Interestingly, nfRas can bind to PI3KC2b, preventing nfRas to bound GTP. ITSN can bind to PI3KC2b, which leads to the release of nfRas and its binding to GTP (Adams et al., 2000; Herrero-Garcia and O’Bryan, 2017).

ITSN is a selective GEF for Cdc42. However, mice deficient in ITSN1 show defects in vesicular transport. Moreover, ITSN binds to RabEP1 (Rab GTPase-binding effector protein), and ITSN overexpression leads to repression of RabEP1-mediated endosomal aggregation and results in the degradation of RabEP1 (Herrero-Garcia and O’Bryan, 2017). Due to its involvement in Rho GTPase activation and its association with the vesicular trafficking function of Rab GTPases, ITSN is a potential link between Cdc42 and Rab GTPases (Figure 3B). We suggest that studying the function of ITSN might provide insight into the interaction between Rho and Rab GTPases during neurodegeneration (Yu et al., 2008; Hunter et al., 2013; Herrero-Garcia and O’Bryan, 2017).

### Interaction between Rab and RhoA GTPases

Similar to Cdc42, RhoA plays a critical role in neuronal migration and regulation of morphological changes in neurons (Hodge and Ridley, 2016). While their roles are similar, these two GTPases differ in the ways they accomplish these functions. RhoA controls cell migration and morphological changes by regulating the assembly of focal adhesions and integrin binding to the extracellular matrix (Brakebusch, 2021). Comparatively, Cdc42 regulates these processes by controlling neuronal actin dynamics (Garvalov et al., 2007). RhoA has also been found to be a potent regulator of axonal elongation, synaptic plasticity, and neuron survival (Stankiewicz and Linseman, 2014; Brakebusch, 2021). RhoA and Rab GTPases are both critical to maintain proper cell movement and the homeostatic environment of neurons (Hall and Lalli, 2010). While regulating these processes, it is possible that an interaction between these small GTPases may be involved in the control of mutual signaling pathways. These signaling pathways could also play a role in neurodegenerative disorders due to the ever-increasing number of studies that have determined the involvement of RhoA and Rab proteins individually in neurodegenerative disorders such as AD and PD.

While there are a number of studies that evaluate the role of Rab proteins and Rho family members in the brain separately, the number of studies that evaluate them together is quite scarce. Nevertheless, overall levels of RhoA and Rab proteins 3A and 5A have been analyzed together in the aging rat brain (Lee et al., 2001). Rab3A and Rab5A levels were found to be unchanged by aging when comparing 2-month-old rats to their 20-month-old counterparts; however, RhoA levels were significantly higher in the aged rats (Lee et al., 2001). Another study of interest pertains to statin use, which has been suggested to reduce the risk of developing AD by up to 70%. In this study Simvastatin, a drug used to treat high cholesterol, significantly reduced Rab4, Rab6, and RhoA in the adult mouse brain (Ostrowski et al., 2016; Figure 3A).

### Interaction between Rab and Rac GTPases

Several studies have shown an interaction between Rab and Rac GTPases through their GEF and GAP proteins. Rac1 has a strong affinity for wild type Rab7, as well as for the constitutively active form of Rab7a. The activated form of Rac1 has also been shown to influence Rab7a activity through its GAP called Armus (Margiotta and Bucci, 2019; Figure 3C). In some neurodegenerative disorders such as ALS, there is evidence that Rab5 and Rac1 reciprocally control each other through the GEF protein Alsin (Topp et al., 2004). The* ALS2* gene that encodes Alsin is mutated in a juvenile form of ALS (Chandran et al., 2007). Interestingly, Alsin was shown to increase Rac1 activity *in vivo* but not *in vitro*, which suggests that there might be a co-factor and or that a posttranslational modification is required for Alsin in order to promote its GEF activity. Alsin can act as a GEF for both Rac1 and Rab5 (Topp et al., 2004; Figure 3C). Mutations in the *rab7a* gene are known to cause the autosomal dominant Charcot-Marie-Tooth type 2B (CMT2B) disease, an axonal peripheral neuropathy (Saveri et al., 2020). Moreover, increased Rac1 and MMP-2 (matrix metalloproteinase-2) activation was found in CMT2B fibroblasts compared to control cells (Romano et al., 2021). These studies suggest that both Rab and Rac are involved in the late stages of the endocytic pathway and play important roles in controlling trafficking and signaling, neurite outgrowth, and neuronal migration. Finally, in the adrenergic N1E-115 neuroblastoma cell line, it is interesting that Rab35 was able to induce neurite growth with the help of Rac1 and Cdc42 but not RhoA (Chevallier et al., 2009; Figure 3B).

### Significance of the Rho-Rab crosstalk in progression of neurodegeneration

Cytoskeletal and intracellular trafficking pathologies are two major features of many neurodegenerative diseases. Thus, it would be significant that the modulators of these pathologies, i.e., Rab and Rho GTPases, physically and/or functionally interact to promote the disease progression. The further elucidation of these potential interactions would call for targeting both Rho and Rab GTPases individually or perhaps in combination. Rab GTPases are widely known to control vesicular transport through endosomal and lysosomal systems. While it is well-established that Rho GTPases control cytoskeletal organization and dynamics, recent research supports a role for Rho GTPases in the spatiotemporal control of intracellular membrane trafficking. Rho GTPases have been recognized to directly influence intracellular membrane sorting and trafficking (Olayioye et al., 2019). Although the contribution of dysregulated Rho GTPase signaling is often focused on the development and progression of cancer, its interactions with Rab GTPases are now emerging in the investigations of neurodegenerative diseases. For instance, there is evidence that modulators of Rho GTPases (such as ITSN, a selective Cdc42 GEF) also participate in the Rab-mediated intracellular trafficking process in neurodegenerative diseases (Wong et al., 2012).

### Future perspectives

Rho and Rab GTPases play crucial roles in modulating neuronal cytoskeleton and vesicular transport, respectively. There is a body of literature emerging showing their potential interconnection physically and functionally. Understanding the crosstalk between Rho and Rab GTPases will provide greater insight into how the cytoskeleton is working together with the vesicular transport system to digest and export cellular waste, promote neuronal survival, and modulate synaptic remodeling. While it is important to apply a reductionist approach to investigate each family of these proteins as independent players, it is time to also consider them as team players trying to accomplish the common goals of maintaining neuronal homeostasis. To achieve better treatment options for neurodegenerative diseases, it is imperative we recognize the ability of neurons to move important components within and outside of the cell, as well as how it uses the cytoskeletal framework to accomplish this goal.

## Author contributions

All authors contributed to the writing of this review and agree to be accountable for the content of the work. All authors contributed to the article and approved the submitted version.
